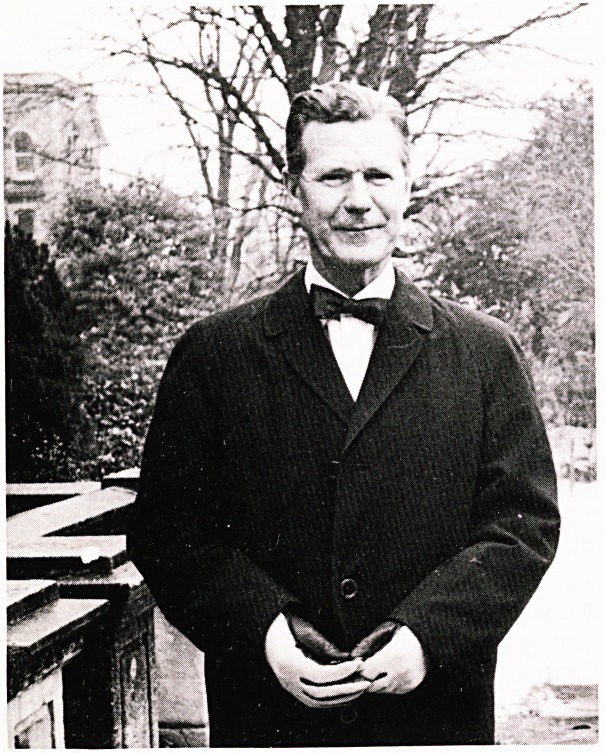# Dr. Clifford D. Evans

**Published:** 1979

**Authors:** 


### Dr. Clifford D. Evans

Dr. Clifford D. Evans, formerly Consultant Dermatologist at the United Bristol Hospitals, died at his home on 14th February 1979 aged 72.

He was educated at Ardingly, Sidney Sussex College, Cambridge and St. Mary's Hospital, where he qualified in 1932. After being a House Physician to the Medical Unit, St. Mary's Hospital and Assistant Medical Officer at Paddington Hospital, he was in General Practice at Keynsham, Somerset from 1933–1939. As a volunteer reserve he entered the RAMC in 1939 and was drafted to France in the 9th General Hospital. After the evacuation from France he commanded the 14th Light Field Ambulance in the Libyan Desert and Italy from 1942–44. In 1944 he became ADMS Second Army Troops BLA landing on D-Day + 1, and after was ADMS (Colonel) at Antwerp and Hamburg until September 1945. He was twice mentioned in dispatches and was awarded the O.B.E.

He had always been interested in Dematology and from 1936–1939 was Assistant Dermatologist at Bath. After the War he became Clinical Assistant to St. John's Hospital for Diseases of the Skin and the Dermatology Department, UCH, and was then appointed temporary Honorary Dermatologist, Bristol Royal Hospital. In December 1946 he became Dermatologist to the Royal United Hospital, Bath, and was appointed Consultant Dermatologist to the United Bristol Hospitals in November 1948.

In spite of the intensive clinical and administrative work in the NHS and in private practice, he found time to undertake laboratory research, particularly in connection with cutaneous carcinogens in patients with turban tumour. He was awarded the M.D. (Cambridge) for his thesis on this subject in 1960. In addition, over the years, he was responsible for considerable clinical research and published many papers on subjects ranging from dermatitis herpetiformis, hedgehog ringworm and reticulo-histiocytosis to contact dermatitis.

However, in the West Country he will be remembered by many patients and General Practitioners. He took enormous care over the management of every patient and had a very loyal and happy relationship with doctors in Bristol and all the surrounding areas. Everyone always had his careful, sympathetic advice. He had a marvellous sense of humour and managed to find a funny side to many problems, for example he kept a collection of amusing letters written to him by General Practitioners and patients — the shortest one being, 'Dear Clifford, Help!'.

He had a personal concern and desire to help with all who came in contact with him including the hospital staff and his colleagues. It was his very careful and gentle approach which endeared him to so many people and made him so successful as an administrator. He served on numerous Hospital, Regional and University Committees — including the Board of Governors, United Bristol Hospitals. His services to Dermatology were acknowledged when, in 1965, he became President of the British Association of Dermatologists and held a very successful meeting in Bristol.

Members of the South West of England and Wales Dermatological Society will always remember his wisdom and kindness, and he enjoyed and valued the companionship and regard of the members of this Society, which he helped to form in 1949.

He was an excellent undergraduate and postgraduate teacher and, apart from the knowledge he imparted, the quiet kindly concern for patients and his sympathy with them, must have inspired countless doctors including junior Dermatologists.

*Continued on page 14*

*Continued from page 12*

He enjoyed all sorts of activities. He rowed for his College 8, much enjoyed sailing, particularly in his early years, and was a keen gardener and golfer. Above all he was a happy companion and had a large circle of real friends — medical and non-medical, who enjoyed being with him. Everyone loved and admired Clifford Evans.

He was married in 1933 to Madge who, with his children Alastair and Sally, survives him. It was always so pleasant to see Clifford and Madge together at meetings and other social occasions, and to savour that happiness, tranquillity and goodness which radiated from them.

## Figures and Tables

**Figure f1:**